# Estimated Artificial Neural Network Modeling of Maximal Oxygen Uptake Based on Multistage 10-m Shuttle Run Test in Healthy Adults

**DOI:** 10.3390/ijerph18168510

**Published:** 2021-08-12

**Authors:** Hun-Young Park, Hoeryoung Jung, Seunghun Lee, Jeong-Weon Kim, Hong-Lae Cho, Sang-Seok Nam

**Affiliations:** 1 Physical Activity and Performance Institute, Konkuk University, 120 Neungdong-ro, Gwangjin-gu, Seoul 05029, Korea; parkhy1980@konkuk.ac.kr; 2 Department of Sports Medicine and Science, Graduate School, Konkuk University, 120 Neungdong-ro, Gwangjin-gu, Seoul 05029, Korea; 3 Department of Mechanical Engineering, Konkuk University, 120 Neungdong-ro, Gwangjin-gu, Seoul 05029, Korea; junghl80@konkuk.ac.kr (H.J.); erioer95@daum.net (S.L.); 4 Graduate School of Professional Therapy, Gachon University, 1342 Seongnam-daero, Sujeong-gu, Seongnam-si 13120, Korea; zeezone@gachon.ac.kr; 5 Inc. Doctor Care Company, Startup Maru Nabi, 48 Buldang 14ro, Seobuk-gu, Cheonan-si 31169, Korea; ily2604@gmail.com; 6 Taekwondo Research Institute of Kukkiwon, 32 Teheran 7-gil, Gangnam-gu, Seoul 06130, Korea

**Keywords:** maximal oxygen uptake, artificial neural network, estimation model, 10 m shuttle run test, graded exercise test

## Abstract

We aimed to develop an artificial neural network (ANN) model to estimate the maximal oxygen uptake (VO_2_max) based on a multistage 10 m shuttle run test (SRT) in healthy adults. For ANN-based VO_2_max estimation, 118 healthy Korean adults (59 men and 59 women) in their twenties and fifties (38.3 ± 11.8 years, men aged 37.8 ± 12.1 years, and women aged 38.8 ± 11.6 years) participated in this study; data included age, sex, blood pressure (systolic blood pressure (SBP), diastolic blood pressure (DBP)), waist circumference, hip circumference, waist-to-hip ratio (WHR), body composition (weight, height, body mass index (BMI), percent skeletal muscle, and percent body), 10 m SRT parameters (number of round trips and final speed), and VO_2_max by graded exercise test (GXT) using a treadmill. The best estimation results (R^2^ = 0.8206, adjusted R^2^ = 0.7010, root mean square error; RMSE = 3.1301) were obtained in case 3 (using age, sex, height, weight, BMI, waist circumference, hip circumference, WHR, SBP, DBP, number of round trips in 10 m SRT, and final speed in 10 m SRT), while the worst results (R^2^ = 0.7765, adjusted R^2^ = 0.7206, RMSE = 3.494) were obtained for case 1 (using age, sex, height, weight, BMI, number of round trips in 10 m SRT, and final speed in 10 m SRT). The estimation results of case 2 (using age, sex, height, weight, BMI, waist circumference, hip circumference, WHR, number of round trips in 10 m SRT, and final speed in 10 m SRT) were lower (R^2^ = 0.7909, adjusted R^2^ = 0.7072, RMSE = 3.3798) than those of case 3 and higher than those of case 1. However, all cases showed high performance (R^2^) in the estimation results. This brief report developed an ANN-based estimation model to predict the VO_2_max of healthy adults, and the model’s performance was confirmed to be excellent.

## 1. Introduction

In the field of sports medicine, cardiorespiratory fitness (CRF) is the most important physical factor in assessing health conditions, including metabolic and cardiovascular disease [1,2]. CRF is a physiological indicator that reflects the ability of the circulatory and respiratory systems to deliver oxygen to skeletal muscles during exercise [3]. CRF is an indicator that reflects risk factors and disease burden throughout an individual’s lifespan, and numerous studies have reported that high levels of CRF reduce morbidity and mortality [3,4,5,6,7]. Additionally, a low CRF increases the risk of obesity, diabetes, hypertension, and cardiovascular disease [8,9,10,11]. As such, CRF can be used as a risk indicator that can provides information in clinical decision making, and efforts to enhance or maintain an appropriate level of CRF via physical activity are critical [1,3,12,13].

Maximal oxygen uptake (VO_2_max) is the main criterion for evaluating CRF, that is, aerobic fitness [1]. VO_2_max is generally measured by indirect calorimetry, which is a method of performing graded exercise tests using a respiratory gas analyzer, heart rate monitor, and treadmill or cycle ergometer in a laboratory [1,14]. In addition, measurement of VO_2_max via indirect calorimetry requires considerable effort, time, money, and a skilled workforce [14,15]. As such, Léger and Lambert [16] developed a 20 m shuttle run test (SRT) using a regression model to conveniently estimate VO_2_max (r = 0.84). Currently, 20 m SRT is widely used to measure cardiovascular fitness, and prediction of VO_2_max using a 20 m SRT has been reported as a useful factor in monitoring the health and physical performance of healthy individuals [1,17,18]. However, the regression model developed by Léger and Lambert [16] has the disadvantage that sex is not considered in the estimated equation, the participants are mainly in their twentieth year of age, and a distance of 25 m (straight line 20 m and safety distance 5 m) or more is required for measurement. Besides, 20 m SRT was originally developed to evaluate the aerobic fitness of athletes, the estimated VO_2_max at the first stage was set at 27.8 mL/kg/min, a high level of exercise intensity. This field test cannot, therefore, be used to evaluate aerobic fitness below a VO_2_max of 27.8 mL/kg/min, and it would impose an excessive, high-risk workload on middle-aged subjects [19]. In previous studies, Matsuzaka et al. [20], Andersen et al. [21], and Mikawa and Senjyu [19] conducted various studies that developed a VO_2_max regression model using 15 m or 20 m SRT; however, efforts to overcome sex, age, and spatial limitations were insufficient. Cho et al. [22] most recently developed the 10 m SRT to evaluate CRF in healthy adults using the regression equation and verified its validity and utility. However, they failed to come up with a regression equation for high regression rates (men: R^2^ = 0.588, women: R^2^ = 0.692).

In addition to regression models, machine learning methods, including artificial neural network (ANN), have been used as regressors to predict VO_2_max with given input parameters including age, sex, and body mass index (BMI) [23,24,25,26]. The ANN-based prediction model, which consists of fully connected sequential layers, can represent not only leaner features but also non-linear features of the target system in virtue of non-linear activation function such as rectified linear unit (ReLU), hyperbolic tangent, and exponential linear unit (ELU). This is the key advantage of the ANN-based prediction model compared to the linear regression model, and it enables the ANN model to be used as a powerful tool, which dramatically changes accessibility of science, research, and practice in all domains [27,28]. Przednowek et al. [1] proposed an ANN-based VO_2_max prediction model using a 20 m SRT and reported that the ANN model provides the most accurate predictions when compared to previous regression models. However, there has not been enough previous reports to assure ANN’s prediction performance to estimate physical fitness data in the field of sports science.

Therefore, the present study proposes an ANN-based prediction model for indirect VO_2_max estimation using a 10 m SRT, which is less spatially constrained than a 20 m SRT. The purpose of our study is to develop estimated ANN modeling of VO_2_max based multistage 10 m SRT, which is easier to perform than the 20 m SRT, and to compare the performance of the ANN-based prediction model with the previous regression models.

## 2. Materials and Methods

### 2.1. Participants

This was a cross-sectional study. The participants of our study were 118 healthy Korean adults (38.3 ± 11.8 years, men aged 37.8 ± 12.1 years (*n* = 59; 15 twenties, 15 thirties, 15 forties, and 14 fifties) and women aged 38.8 ± 11.6 years (*n* = 59; 15 twenties, 15 thirties, 15 forties, and 14 fifties)), who were selected via random sampling and who were non-smokers and had no history of musculoskeletal, cardiovascular, or pulmonary diseases or diabetes. All study participants were provided with a detailed explanation of the purpose and process of the present study, and consent was obtained after being fully informed about the possible side effects related to the experiment before the start of the present study. The study was reviewed and approved by the Institutional Review Board of KyungHee University (KHU IRB 2014-G01) and conducted in accordance with the Declaration of Helsinki.

**Figure 1:**
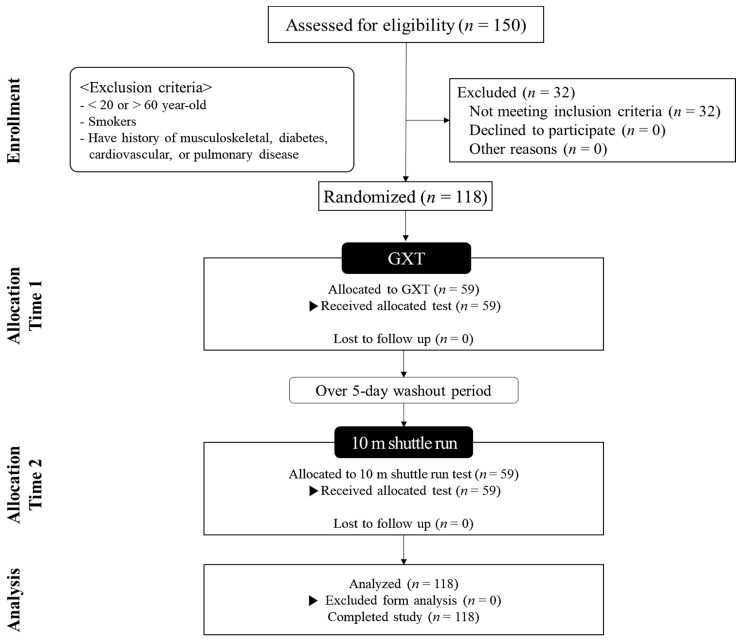
Consolidated Standards of Reporting Trials (CONSORT) flow diagram. GXT, graded exercise test.

The characteristics of the participants are presented in Table 1, and the Consolidated Standards of Reporting Trials flow diagram is shown in Figure 1.

### 2.2. Study Design

**Figure 2:**
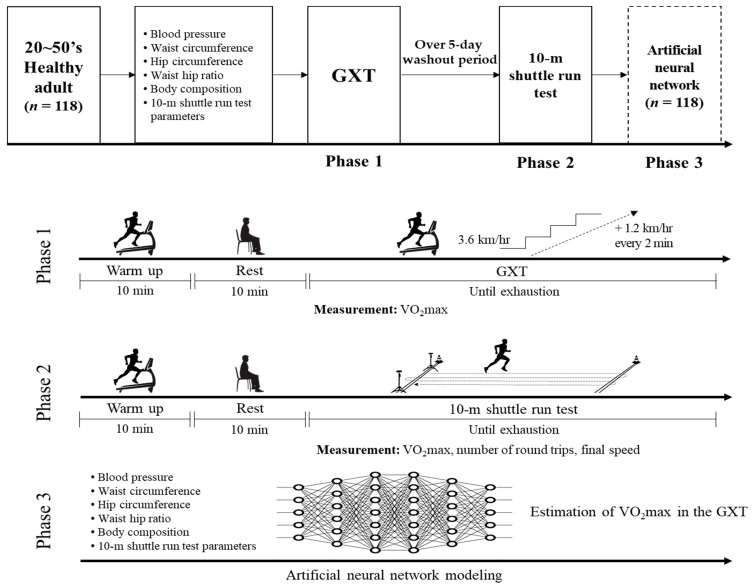
Study design. GXT, graded exercise test; VO_2_max, maximal oxygen uptake.

The design of the present study is shown in Figure 2. We recruited 150 healthy adult volunteers (75 men and 75 women) in healthy adults. Subsequently, a questionnaire test using the Physical Activities Readiness Questionnaire and You and American Heart Association/American College of Sports Medicine Health/Fitness Facility Pre-Participation Screening Questionnaire and blood pressure (BP) test were performed. Additionally, cardiovascular disease risk in volunteers was classified using the criteria suggested by the American Association of Cardiovascular and Pulmonary Rehabilitation. Thirty-two volunteers with risk factors for participation in the exercise test were excluded through the screening process, and 118 volunteers participated in this study. The selected 118 participants were first subjected to waist circumference, hip circumference, waist-to-hip ratio (WHR), and body composition test. The graded exercise test (GXT) and multistage 10 m SRT were performed sequentially with an interval of at least 5 days. All participants underwent GXT using a treadmill (Precor 932i, Precor, WA, USA), an automatic respiration metabolic analyzer K4B2 (Cosmed, Rome, Italy), and a heart rate monitor (S610i, Polar, Helsinki, Finland). The GXT protocol started at 3.6 km/h and increased the speed by 1.2 km/h every 2 min, and the treadmill slopes were set to 0% at all stages [26]. The 10 m SRT was measured using an automatic respiration metabolic analyzer K4B2 (Cosmed, Rome, Italy) and heart rate monitor (S610i, Polar, Helsinki, Finland) along with nine sound sources composed of scale (Do, Re, Mi, Fa, So, La, Ti, Do) and buzzer sounds. The detailed protocol of the 10 m SRT is shown in Table 2.

After all measurements were completed, ANN modeling was performed to estimate the VO_2_max measured by GXT using the easy-to-measure dependent parameters acquired via data including age; sex; BP (systemic blood pressure (SBP), diastolic pressure (DBP)); waist circumference; hip circumference; WHR; body composition (height, weight, BMI, percent skeletal muscle, and percent body); 10 m SRT parameters (number of round trips in 10 m SRT and final speed in 10 m SRT). For this process, the acquired data were used to train an estimated ANN model, and 30% of the data was randomly sampled from the training dataset for the validity test.

### 2.3. Blood Pressure

After the participants sufficiently rested for >20 min, BP of the right brachial artery was measured twice using an autonomic BP monitor (HBP-9020, Omron, Tokyo, Japan), and the average value was used as the result value.

### 2.4. Waist Circumference, Hip Circumference, and WHR

Waist circumference and hip circumference were measured using a tape measure (Balzer 80206F, Hoechstmass, Sulzbach, Germany). The waist circumference was measured at the level of the navel, and the hip circumference was measured at the greatest protrusion. Moreover, the WHR was calculated by dividing the waist circumference (cm) by the hip circumference (cm).

### 2.5. Body Composition

The body composition parameters (height, weight, BMI, percent skeletal muscle, and percent body) of all the participants were analyzed using a stadiometer (YM-1, KDS, Seoul, Korea) and bioelectrical impedance analyzer (Karada scan, Omron, Kyoto, Japan). All participants wore lightweight clothing and were asked to remove all metal items on their body.

### 2.6. Graded Exercise Test

The GXT was performed using a treadmill (Precor 932i, Precor, WA, USA), an automatic respiration metabolic analyzer K4B2 (Cosmed, Rome, Italy), and a heart rate monitor (S610i, Polar, Helsinki, Finland). The GXT protocol started at 3.6 km/h and increased the speed by 1.2 km/h every 2 min, and the treadmill slopes were set to 0% at all stages [19]. Respiratory gas data (minute ventilation, oxygen uptake, carbon dioxide excretion, and respiratory exchange ratio and heart rate (HR)) were measured every 10 s during GXT, and blood lactate levels were analyzed using a Lactate pro2 (Arkray, Kyoto, Japan) by collecting 80 μL of blood from the capillaries in the fingertips every min during GXT. When at least three of the following conditions were fulfilled while performing GXT, it was judged to have reached the stage of exhaustion, and the exercise was terminated, and VO_2_max, the result value, was acquired: (1) when the HR did not increase in proportion to exercise intensity, (2) when VO_2_ did not increase, even when exercising intensity increased, (3) when the respiratory exchange ratio (RER) was 1.10 or higher, (4) when the Borg scale score was 17 or higher, and (5) when the predicted HRmax exceeded 90% HRmax.

### 2.7. Ten-Meter Shuttle Run Test

The 10 m SRT was performed using an automatic respiration metabolic analyzer K4B2 (Cosmed, Rome, Italy) and heart rate monitor (S610i, Polar, Helsinki, Finland), and the speed was controlled by nine sound sources (Do, Re, Mi, Fa, So, La, Ti, Do) and a buzzer sound. The 10-SRT adjusts the speed of by nine sound sources, providing incremental exercise load similar to the GXT. Therefore, we converted the speed of each step in the step-by-step 10 m SRT to beats per min and adjusted the playback speed of the sound accordingly. The 10 m SRT also checked the oxygen uptake and HR like the GXT, and in consideration of the characteristics of the 10 m SRT, the test was terminated when the participant could not repeatedly follow the speed of the sound source. The result value obtained via the 10 m SRT corresponded to VO_2_max, the number of round trips, and the final speed.

### 2.8. Statistical Analysis

All statistical analyses were conducted using SPSS version 25.0 (IBM Corp., Armonk, NY, USA) for Windows. Data are presented as mean ± standard deviation. The normality of the distribution of all outcome variables was verified using the Shapiro–Wilk test. A paired *t*-test was used to verify the difference between VO_2_max in the GXT and VO_2_max in the 10 m SRT. Pearson’s correlation analysis was used to analyze the correlation between dependent parameters and VO_2_max measured by GXT and the correlation between the measured VO_2_max and the estimated VO_2_max. Statistical significance was set at *p* < 0.05.

### 2.9. Artificial Neural Network-Based Prediction Model

An ANN, which is a fully connected feedforward network composed of three different types of layers, that is, the input layer, hidden layer, and output layer, was used to construct the VO_2_max prediction models. The input layer consisted of 14 nodes (independent variables) and received predictors to estimate VO_2_max. The input variables were age; sex; blood pressure (SBP, DBP); waist circumference; hip circumference; WHR; body composition (weight, height, BMI, percent skeletal muscle, and percent body fat); 10 m SRT parameters (number of round trips and final speed). These input data are standardized so that the average and standard deviation of all input data are equal to 0 and 1, respectively, to prevent biased output to the specific input parameter. The standardized input data are passed to the hidden layers, which are composed of three layers with 21 nodes (a total of 63 nodes), and the final estimation of VO_2_max by GXT is given through the single node output layer. The proposed prediction model is a multiple-input single-output type. The 10 m SRT data gathered from the participants were used to train the proposed ANN-based prediction model. The Elu function is used as an activation function for all the nodes in the hidden layer. The batch normalization and dropout techniques are applied in the training of the ANN model to prevent overfitting. The training was conducted with a learning rate of 0.001 via stochastic gradient descent until the number of epochs reached 1000. The proposed ANN model was implemented using the Python (3.8.5) language using Tensorflow (2.4.1) and Keras (2.4.3) packages.

## 3. Results

### 3.1. Estimation Accuracy of Artificial Neural Network-Based Maximal Oxygen Uptake Prediction Model

We verified the absolute value of the standardized residual as ≥3 to omit the outlier data. There were no outlier data in the ANN-based VO_2_max using the GXT estimation model. The correlation between the measured VO_2_max and the dependent variables (input data) is shown in Table 3.

In total, 118 sets of SRT data were used to train the ANN-based VO_2_max estimation model, and 36 sets of data randomly sampled from the training datasets were used as test data to evaluate the estimation accuracy of the proposed model. Table 4 shows the estimation accuracy of the ANN-based VO_2_max prediction model. The VO_2_max measured by GXT was estimated using the ANN-based prediction model by varying the input data of the model. In case 1, six input data composed of age, sex, height, weight, BMI, number of round trips in 10 m SRT, and final speed in 10 m SRT were used to estimate the VO_2_max values: the number of input data increased in cases 2 (age, sex, height, weight, BMI, waist circumference, hip circumference, WHR, number of round trips in 10 m SRT, and final speed in 10 m SRT) and 3 (age, sex, height, weight, BMI, waist circumference, hip circumference, WHR, SBP, DBP, number of round trips in 10 m SRT, and final speed in 10 m SRT) to investigate the effects of the inputs on the estimation accuracy. The estimation accuracy of the ANN-based VO_2_max prediction model was evaluated using R^2^, adjusted R^2^, and root mean square error (RMSE).

In the estimation models of measured VO_2_max by GXT, the best estimation result (R^2^ = 0.8206, adjusted R^2^ = 0.7010, RMSE = 3.1301) was obtained in case 3, while the worst result (R^2^ = 0.7765, adjusted R^2^ = 0.7206, RMSE = 3.494) was obtained in case 1. Case 2 (R^2^ = 0.7909, adjusted R^2^ = 0.7072, RMSE = 3.3798) showed lower estimation results than case 3 and higher estimation results than case 1. However, all the cases showed high performance in the estimation results.

### 3.2. Difference between Measured Maximal Oxygen Uptake and Artificial Neural Network-Based Predicted Maximal Oxygen Uptake

**Figure 3:**
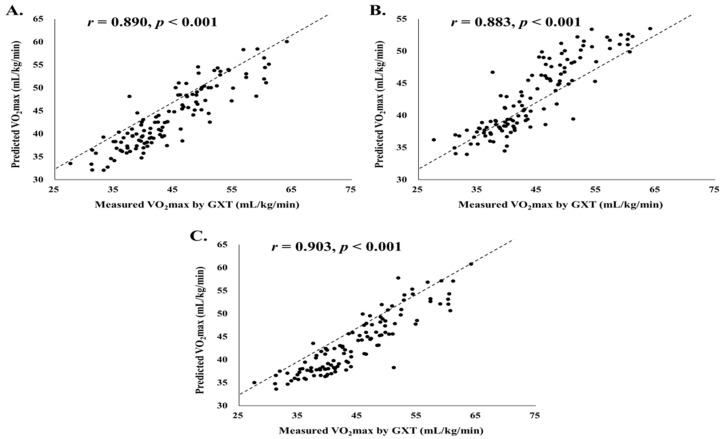
Scatter plot between measured VO_2_max by GXT and the estimated VO_2_max by case 1, case 2, and case 3 of healthy adults. (**A**). Case 1 scatter plot and correlation. (**B**). Case 2 scatter plot and correlation. (**C**). Case 3 scatter plot and correlation. GXT, graded exercise test; VO_2_max, maximal oxygen uptake.

In the present study, there was no significant difference between the measured VO_2_max by GXT and estimated VO_2_max in case 1; however, there was a significant difference between VO_2_max by GXT and the estimated VO_2_max by cases 2 and 3 (Table 5). The mean bias between the measured VO_2_max and the estimated VO_2_max in cases 1, 2, and 3 were −0.54 mL/kg/min, −1.32 mL/kg/min, and −0.93 mL/kg/min, respectively (Table 5). The measured and estimated VO_2_max in all cases showed a similar average value, and their correlation coefficients also demonstrated a significant correlation (measured VO_2_max and estimated VO_2_max in case 1: *r* = 0.890, *p* < 0.001; measured VO_2_max and estimated VO_2_max in case 2: *r* = 0.883, *p* < 0.001; measured VO_2_max and estimated VO_2_max in case 3: *r* = 0.903, *p* < 0.001) (Figure 3).

## 4. Discussion

Our brief report was conducted to develop an ANN-based estimation model for predicting the VO_2_max of healthy adults using 10 m SRT parameters (number of round trips in 10 m SRT and final speed in 10 m SRT) and various easy-to-measure dependent variables (age, sex, height, weight, BMI, waist circumference, hip circumference, WHR, SBP, and DBP). Based on the data obtained, we developed three cases of ANN-based VO_2_max estimation models (case 1: R^2^ = 0.7765, adjusted R^2^ = 0.7206, RMSE = 3.494, case 2: R^2^ = 0.7909, adjusted R^2^ = 0.7072, RMSE = 3.3798, case 3: R^2^ = 0.8206, adjusted R^2^ = 0.7010, RMSE = 3.1301).

VO_2_max is the main criterion for evaluating CRF, that is, aerobic fitness [1], and is a strong health indicator [1,2,3]. Previous studies have reported that high levels of CRF reduce morbidity and mortality, and low CRF levels increase the risk of obesity, diabetes, hypertension, and cardiovascular disease [3,4,5,6,7,8,9,10,11]. Therefore, the easy and convenient measurement of VO_2_max is critical for evaluating CRF [1,14,15]. Thus, many researchers have tried various methods to easily and conveniently estimate VO_2_max, and a regression model for estimating VO_2_max using a 20 m SRT has been commonly used [16,17,18,19,20,21]. In this regard, Léger and Lambert [16] first validated the 20 m SRT using a regression model for conveniently estimated VO_2_max in 91 adults (32 women and 59 men, aged 27.3 ± 9.2 and 24.8 ± 5.5 years, respectively) and with mean VO_2_max (39.3 ± 8.3 and 51.6 ± 7.8 mL/kg/min, respectively). Consequently, they reported the following regression model: VO_2_max = 5.857 × final speed in 10 m SRT − 19.458, *r* = 0.84, and SEE = 5.4 and concluded that a 20 m SRT is a valid and reliable test for the estimation of VO_2_max in male and female adults. Silva et al. [29] verified two models to estimate VO_2_max in 104 Portuguese youths (54 girls and 60 boys), aged 10–18 years, using a 20 m SRT, and recommended the following multiple linear regression model: VO_2_max = 43.313 + 4.567 × sex − 0.560 × BMI + 2.785 × stage; *r* = 0.84, SEE = 4.9. Matsuzaka et al. [20] examined the validity of a 20 m SRT as an aerobic fitness test for young adults (56 men and 99 women aged 18–23 years) and reported the following regression model: 1. VO_2_max = −2.19 − 3.46 × sex − 0.416 × BMI + 5.22 × maximal running speed (R^2^ = 0.88, SEE = 3.0 mL/kg/min), 2. VO_2_max = 42.4 − 2.85 × sex − 0.488 × BMI + 0.247 × total laps (R^2^ = 0.88, SEE = 3.0 mL/kg/min). These previous studies confirm that estimation of VO_2_max using multiple regression equations is a suitable method.

Recently, modified SRT with a reduced distance has been developed to simplify measurements and reduce spatial constraints. Takuro et al. [30] investigated the reproducibility of 15 m SRT in 25 healthy adults and concluded that a 15 m SRT provides reproducible results for the assessment of exercise capacity in healthy young participants. Mikawa and Senjyu [19] developed a modified SRT, the 15 m SRT, to assess CRF (VO_2_max) using distance and exercise time in middle-aged adults (*n* = 68) and reported the following two regression models: (1) VO_2_max = 14.56 + 0.02 × 15-m SRT distance (*r* = 0.86, *p* < 0.01), (2) VO_2_max = 80.17 − 0.057 × 15-m SRT time (*r* = −0.51, *p* < 0.01). Based on these results, they insisted that the 15 m SRT is valid and safe for evaluating VO_2_max and is recommended as a field test for evaluating aerobic fitness in middle-aged adults.

In the present brief report, we developed a 10 m SRT for more convenient and practical CRF measurements in healthy adults and developed a predictive model based on ANN with various easy-to-measure dependent variables (age, sex, height, weight, BMI, waist circumference, hip circumference, WHR, SBP, DBP, number of round trips in 10 m SRT, and final speed in 10 m SRT) for more accurate and reliable prediction of VO_2_max. Our ANN-based modeling of VO_2_max based on 10 m SRT in healthy adults showed better performance and accuracy (higher R^2^ and lower SEE) even though it is easier to measure than 15 m or 20 m estimation models using the regression equation. In particular, Cho et al. [22] developed the 10 m SRT in healthy adults (*n* = 120) to improve the spatial limitation by reducing the measurement to a 10 m distance and to resolve the bias uniform distributions of sex and age. They reported the following regression model: 1. VO_2_max (men) = 0.231 × 10 m SRT count − 1.975 × 10 m SRT final speed − 0.073 × BMI − 23.488 × WHR + 60.909 (R^2^ = 0.588, SEE = 4.17 mL/kg/min), 2. VO_2_max (women) = 0.272 × 10 m SRT count − 1.530 × 10 m SRT final speed − 0.649 × BMI + 15.392 × WHR − 0.029 × age + 30.345 (R^2^ = 0.692, SEE = 3.39 mL/kg/min). Compared with the study by Cho et al. [22], which developed a VO_2_max estimation model using the same 10 m SRT based on similar participants (twenties and fifties healthy adults) and sample sizes (*n* = 120), our ANN-based VO_2_max modeling is an accurate and valid method for estimating VO_2_max. Moreover, considering the high positive correlation (case 1: *r* = 0.890, *p* < 0.001; case 2: *r* = 0.883, *p* < 0.001; case 3: *r* = 0.903, *p* < 0.001) between the measured VO_2_max by GXT and estimated VO_2_max in all models of the three cases we developed, we can confirm that our ANN-based modeling shows high validity and reliability. Therefore, our estimated ANN-based VO_2_max modeling-based multistage 10 m SRT can be effectively utilized to evaluate CRF, the most important factor for estimating adult health conditions, including metabolic and cardiovascular disease.

In the present study, we presented that 10 m SRT with an ANN-based estimation model can be used to predict VO_2_max in easy and reliable manner, but there remains a limitation that should be resolved in future research. Since the number of samples (118 samples) used to train an ANN-based estimation model is too small to represent general VO_2_max characteristics, large-scale trials covering more than hundreds of subjects should be performed to secure enough training dataset and more rigorous performance validation of the estimation model. Additionally, we believe that the performance, validity, and reliability of the ANN-based estimation model between 10 m SRT and other simple tests estimating VO_2_max should be compared.

## 5. Conclusions

In our brief report, we developed ANN-based estimation model to predict the VO_2_max based on multistage 10 m SRT in healthy adults, and the model’s performance was confirmed to be excellent. Therefore, we believe that ANN-based modeling of VO_2_max based on multistage 10 m SRT is a highly scalable, effective, validated, and reliable method for predicting CRF in healthy adults.

## Figures and Tables

**Table 1:** Participants’ characteristics.

	Both (*n* = 118)	Men (*n* = 59)	Women (*n* = 59)
Age (yrs)	38.29 ± 11.82	37.75 ± 12.09	38.83 ± 11.62
Height (cm)	166.69 ± 8.16	172.69 ± 6.19	160.69 ± 4.77
Weight (kg)	65.36 ± 10.72	72.99 ± 8.61	57.73 ± 6.26
BMI (kg/m^2^)	23.41 ± 2.54	24.44 ± 2.24	22.37 ± 2.40
Percent skeletal muscle (%)	29.86 ± 3.76	32.89 ± 2.42	26.84 ± 2.01
Percent body fat (%)	24.42 ± 5.56	20.97 ± 4.51	27.87 ± 4.22
SBP (mmHg)	121.89 ± 9.99	126.61 ± 7.07	117.17 ± 10.30
DBP (mmHg)	79.14 ± 10.05	82.85 ± 9.32	75.44 ± 9.43
Waist circumference (cm)	81.32 ± 7.66	84.38 ± 7.70	78.26 ± 6.33
Hip circumference (cm)	96.04 ± 5.16	96.62 ± 5.52	95.47 ± 4.75
WHR	0.85 ± 0.05	0.87 ± 0.05	0.82 ± 0.04
Numbers of round trips in 10 m SRT (n)	115.61 ± 26.19	131.49 ± 24.08	99.73 ± 17.06
Final speed in 10 m SRT (km/h)	10.03 ± 0.94	10.56 ± 0.89	9.50 ± 0.64
VO_2_max by GXT (mL/kg/min)	44.27 ± 7.70	49.08 ± 6.70	39.46 ± 5.26

Note: Values are expressed as the mean ± standard deviation. BMI, body mass index; SBP, systolic blood pressure; DBP, diastolic blood pressure; WHR, waist-to-hip ratio; SRT, shuttle run test; VO_2_max, maximal oxygen uptake; GXT, graded exercise test.

**Table 2:** The 10 m shuttle run test protocol.

Start Time (min)	Finish Time (min)	Speed (km/h)	Moving Time Per 10 m (s)	Beats Per min	Number of Shuttles
0:00:00	0:01:00	3.6	10.00	54	6
0:01:00	0:02:00	4.8	7.50	72	8
0:02:00	0:03:00	6.0	6.00	90	10
0:03:00	0:04:00	6.0	6.00	90	10
0:04:00	0:05:00	7.2	5.00	108	12
0:05:00	0:06:00	7.2	5.00	108	12
0:06:00	0:07:00	8.4	4.29	126	14
0:07:00	0:08:00	8.4	4.29	126	14
0:08:00	0:09:00	9.6	3.75	144	16
0:09:00	0:10:00	9.6	3.75	144	16
0:10:00	0:11:00	10.8	3.33	162	18
0:11:00	0:12:00	10.8	3.33	162	18
0:12:00	0:13:00	12.0	3.00	180	20
0:13:00	0:14:00	12.0	3.00	180	20
0:14:00	0:15:00	13.2	2.73	198	22
0:15:00	0:16:00	13.2	2.73	198	22
0:16:00	0:17:00	14.4	2.50	216	24
0:17:00	0:18:00	14.4	2.50	216	24
0:18:00	0:19:00	15.6	2.31	234	26
0:19:00	0:20:00	15.6	2.31	234	26
0:20:00	0:21:00	16.8	2.14	252	28
0:21:00	0:22:00	16.8	2.14	252	28

**Table 3:** Correlation between dependent variables (input data) and measured VO_2_max by GXT (output data).

	VO_2_max by GXT			
		Total	Men	Women
Age (yrs)	Correlation	−0.339 *	−0.391 *	−0.412 *
	*p*-value	0.000	0.002	0.001
Height (cm)	Correlation	0.412 *	−0.182	0.047
	*p*-value	0.000	0.167	0.724
Weight (kg)	Correlation	0.215 *	−0.440 *	−0.409 *
	*p*-value	0.019	0.000	0.001
BMI (kg/m^2^)	Correlation	−0.047	−0.431 *	−0.437 *
	*p*-value	0.617	0.001	0.001
Percent skeletal muscle (%)	Correlation	0.767 *	0.593 *	0.526 *
	*p*-value	0.000	0.000	0.000
Percent body fat (%)	Correlation	−0.783 *	−0.697 *	−0.577 *
	*p*-value	0.000	0.000	0.000
SBP (mmHg)	Correlation	0.194 *	−0.198	−0.125
	*p*-value	0.036	0.133	0.347
DBP (mmHg)	Correlation	0.066	−0.300 *	−0.145
	*p*-value	0.477	0.021	0.275
Waist circumference (cm)	Correlation	−0.151	−0.642 *	−0.441 *
	*p*-value	0.104	0.000	0.000
Hip circumference (cm)	Correlation	−0.272 *	−0.457 *	−0.419 *
	*p*-value	0.003	0.000	0.001
WHR	Correlation	0.003	−0.561 *	−0.277 *
	*p*-value	0.974	0.000	0.034
Numbers of round trips in 10 m SRT (*n*)	Correlation	0.837 *	0.764 *	0.688 *
	*p*-value	0.000	0.000	0.000
Final speed in 10 m SRT (km/h)	Correlation	0.777 *	0.683 *	0.611 *
	*p*-value	0.000	0.000	0.000

Note: Values are expressed as the mean ± standard deviation. Significant correlation between measured VO_2_max by GXT and dependent variables, * *p* < 0.05. BMI, body mass index; SBP, systolic blood pressure; DBP, diastolic blood pressure; WHR, waist-to-hip ratio; SRT, shuttle run test; VO_2_max, maximal oxygen uptake; GXT, graded exercise test.

**Table 4:** Estimation accuracy of artificial neural network-based maximal oxygen uptake estimation model for three cases of various input compositions.

	Measured VO_2_max by GXT		
	R^2^	Adjust R^2^	RMSE
Case 1 ANN-based estimation	0.7765	0.7206	3.4940
Case 2 ANN-based estimation	0.7909	0.7072	3.3798
Case 3 ANN-based estimation	0.8206	0.7010	3.1301

Note: The inputs of case 1 consisted of age, sex, height, weight, BMI, number of round trips in the 10 m shuttle run test, and final speed in the 10 m shuttle run test. The inputs of case 2 consist of inputs of case 1, waist circumference, hip circumference, and waist-hip ratio (WHR), and the inputs of case 3 consist of inputs of case 2, SBP, and DBP. VO_2_max, maximal oxygen uptake; GXT, graded exercise test; ANN, artificial neural network; RMSE, root mean square error.

**Table 5:** Measured and estimated VO_2_max in all cases of healthy adults.

Model		Mean ± S.D.	Bias	*t*-Value	*p*-Value
Case1	Predicted treadmill VO_2_max (mL/kg/min)	43.73 ± 6.62	−0.54	1.674	0.097
	Measured treadmill VO_2_max (mL/kg/min)	44.27 ± 7.70			
Case2	Predicted treadmill VO_2_max (mL/kg/min)	42.94 ± 5.55	−1.32	3.753	0.000
	Measured treadmill VO_2_max (mL/kg/min)	44.27 ± 7.70			
Case3	Predicted treadmill VO_2_max (mL/kg/min)	43.33 ± 6.36	−0.93	3.012	0.003
	Measured treadmill VO_2_max (mL/kg/min)	44.27 ± 7.70			

Note: VO_2_max, maximal oxygen uptake.

## Data Availability

The data presented in this study are available on request from the corresponding author.
